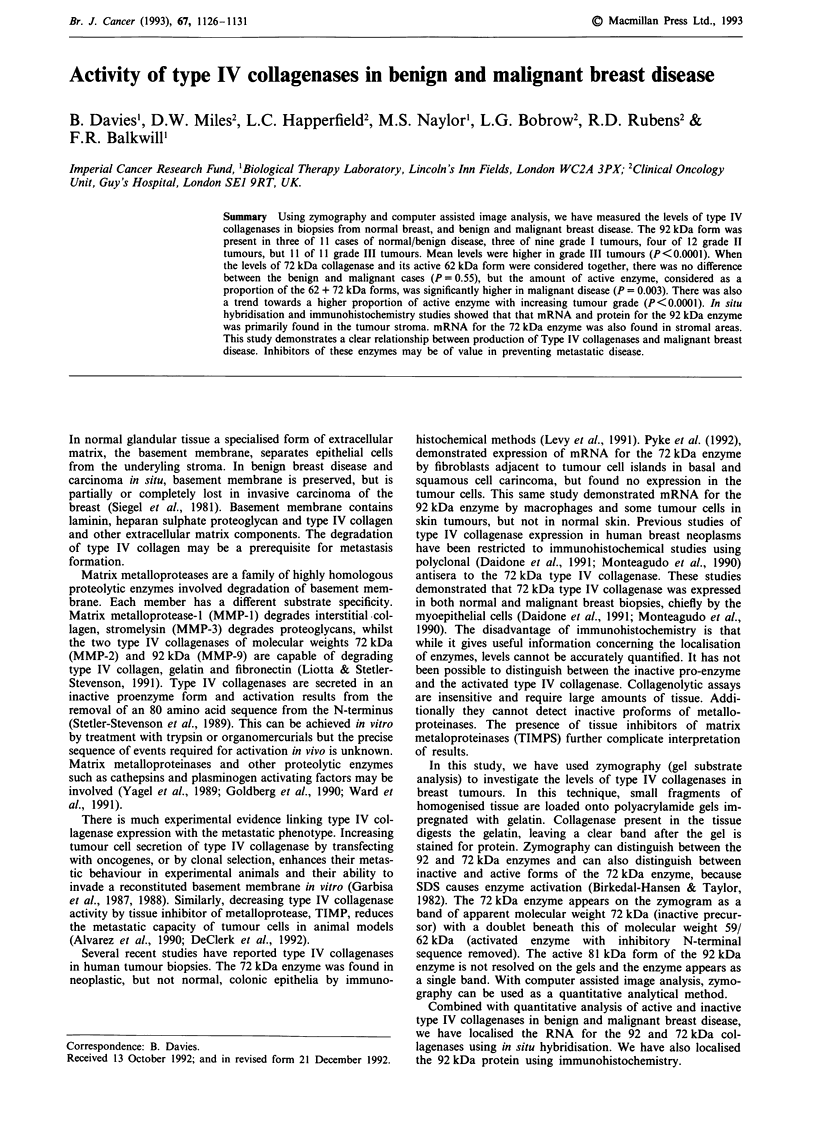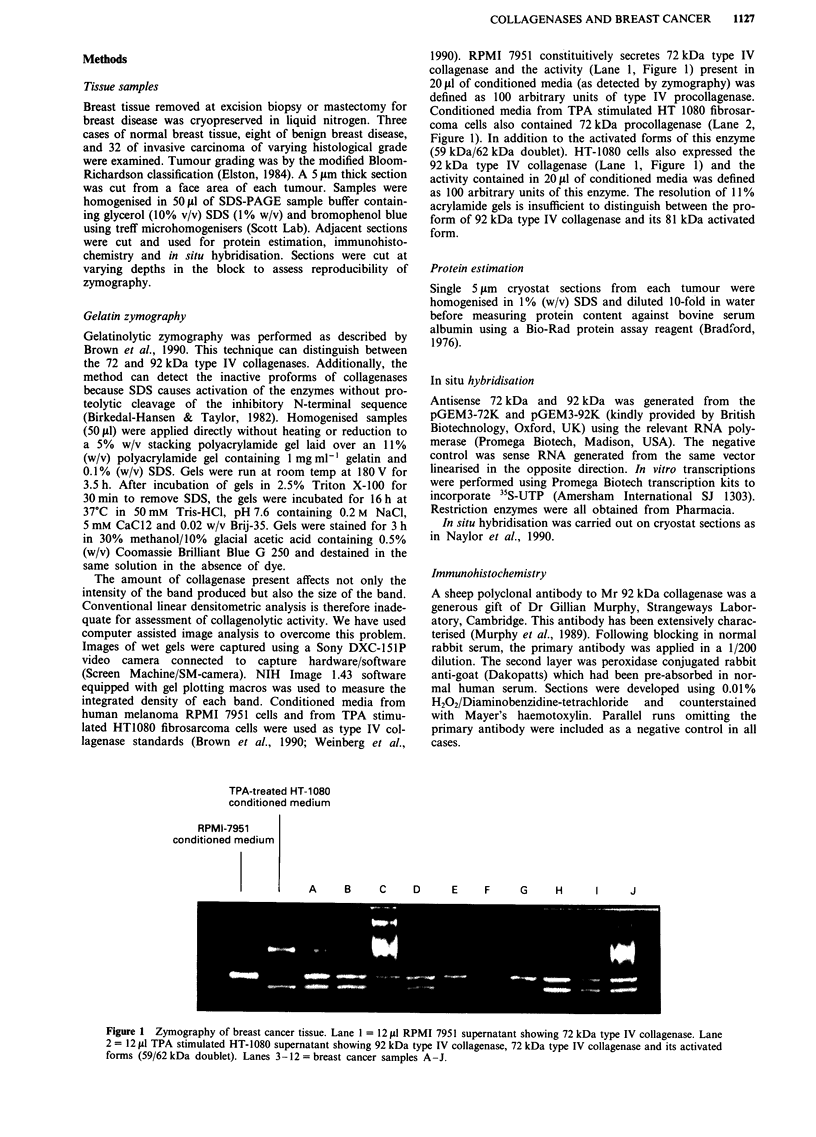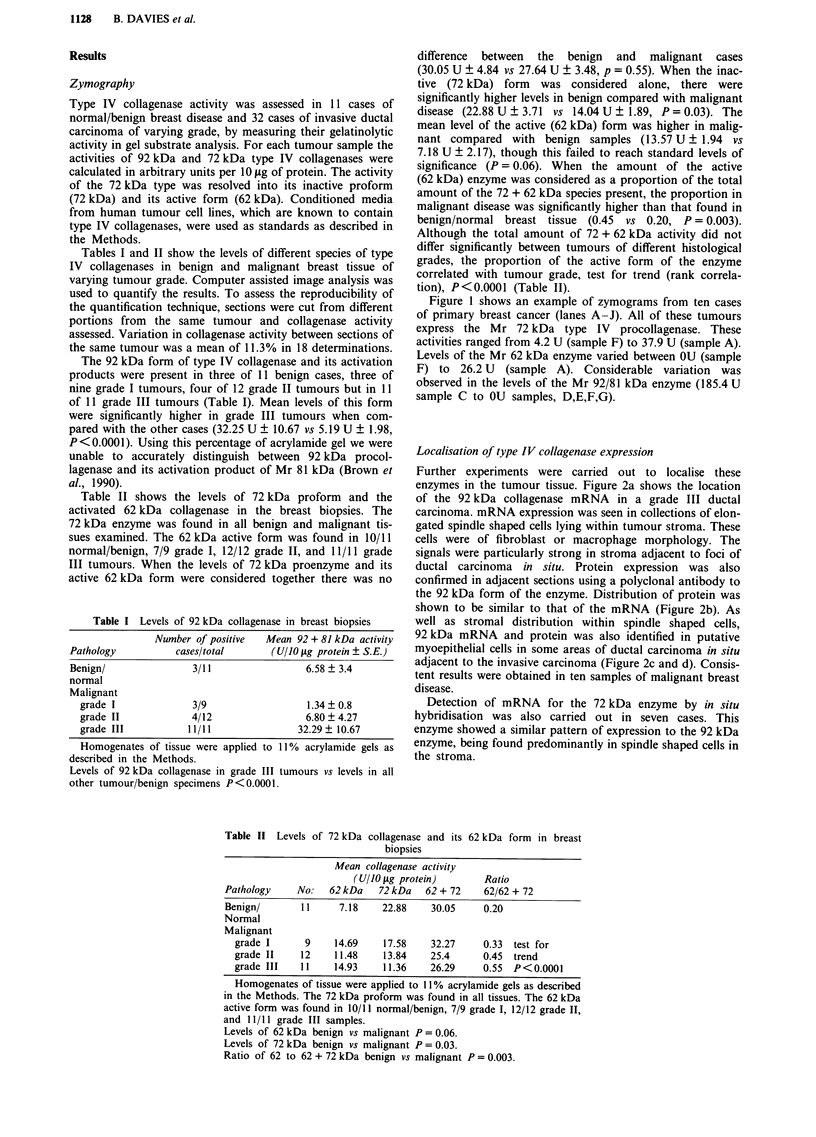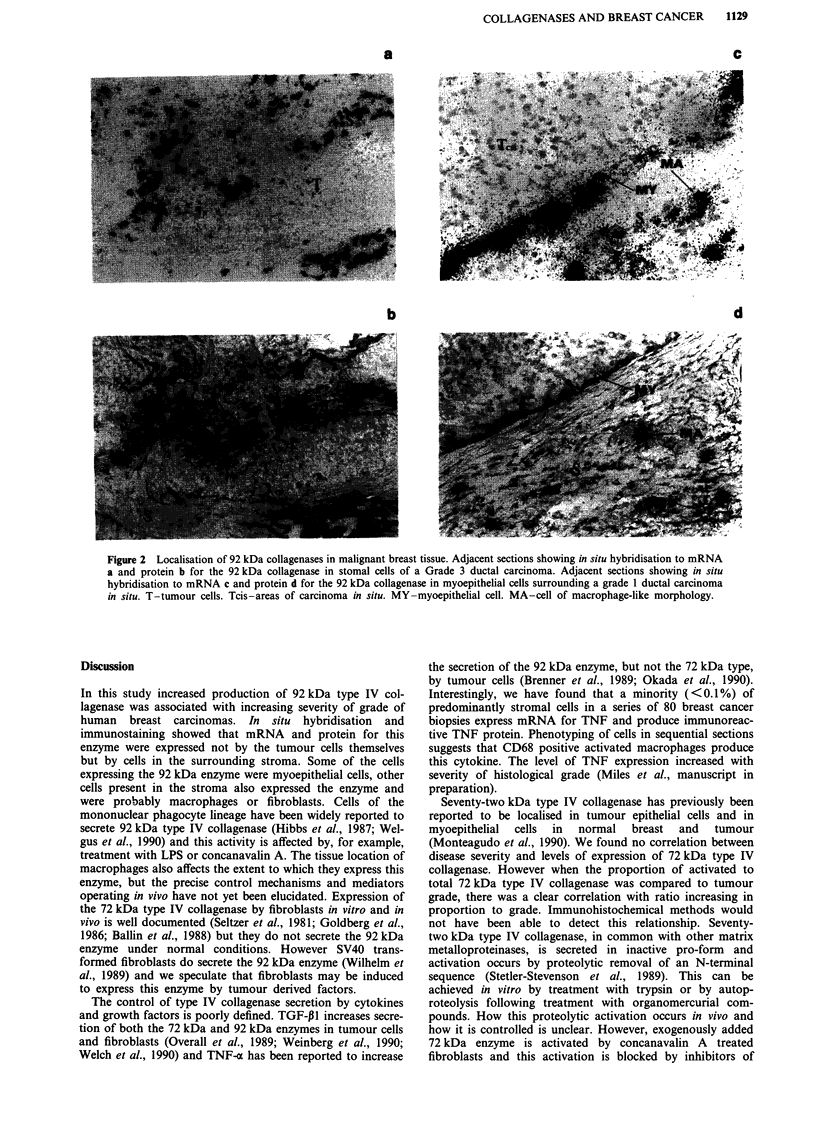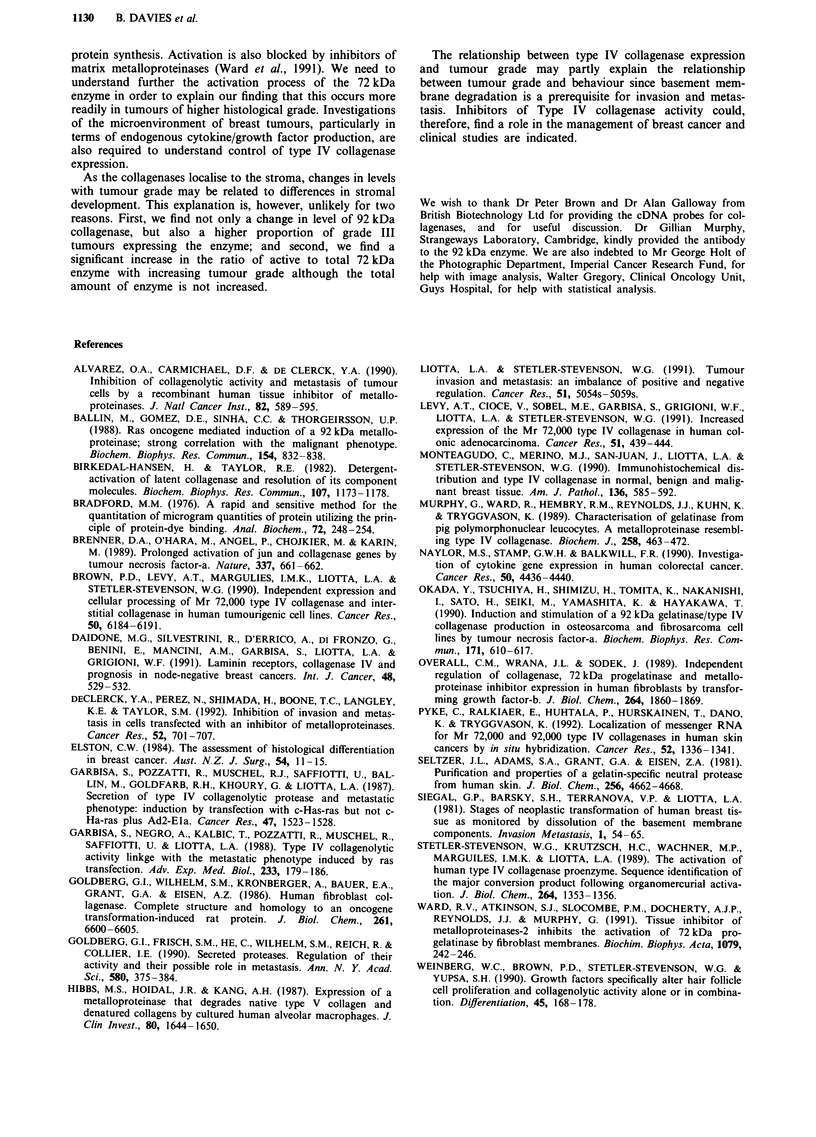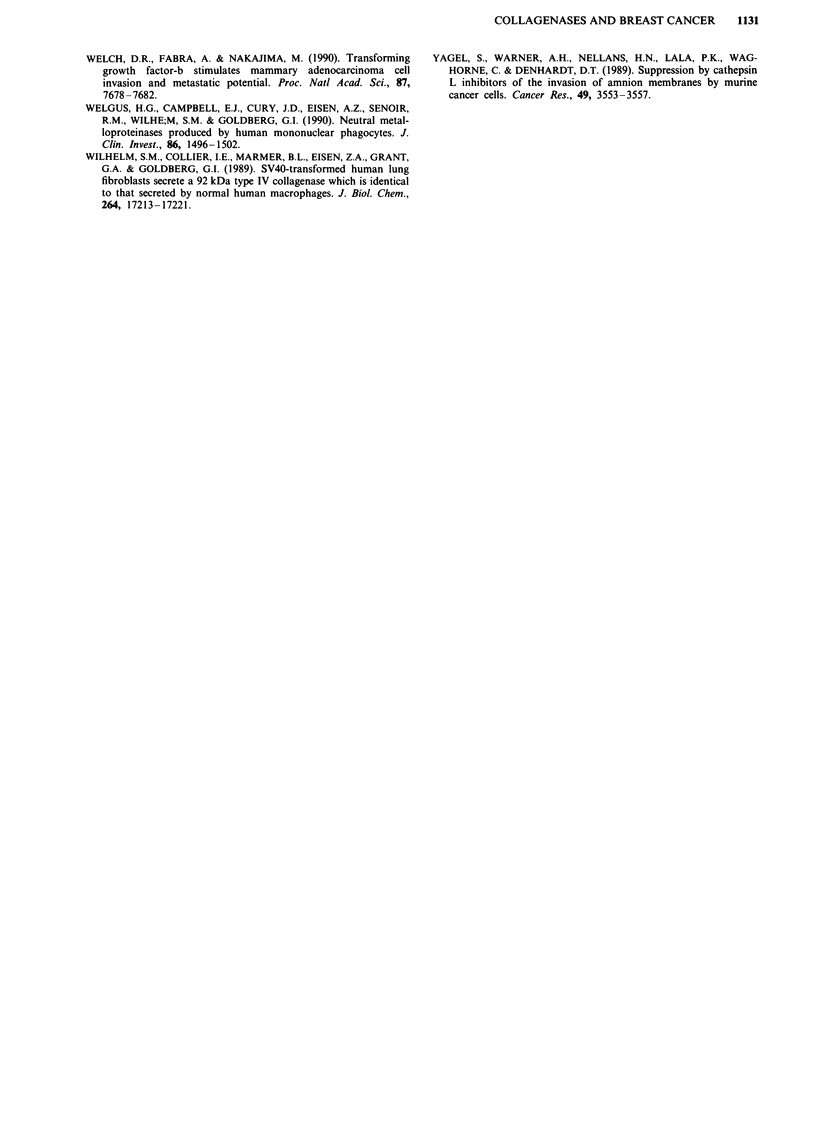# Activity of type IV collagenases in benign and malignant breast disease.

**DOI:** 10.1038/bjc.1993.207

**Published:** 1993-05

**Authors:** B. Davies, D. W. Miles, L. C. Happerfield, M. S. Naylor, L. G. Bobrow, R. D. Rubens, F. R. Balkwill

**Affiliations:** Imperial Cancer Research Fund, Biological Therapy Laboratory, London, UK.

## Abstract

**Images:**


*Br. J. Cancer* (1993), **67**, 1126–1131 © Macmillan Press Ltd., 1993

### Activity of type IV collagenases in benign and malignant breast disease

B. Davies[1], D.W. Miles[2], L.C. Happerfield[2], M.S. Naylor[1], L.G. Bobrow[2], R.D. Rubens[2] & F.R. Balkwill[1]

*Imperial Cancer Research Fund, [1]Biological Therapy Laboratory, Lincoln's Inn Fields, London WC2A 3PX; [2]Clinical Oncology Unit, Guy's Hospital, London SE1 9RT, UK.*

**Summary** Using zymography and computer assisted image analysis, we have measured the levels of type IV collagenases in biopsies from normal breast, and benign and malignant breast disease. The 92 kDa form was present in three of 11 cases of normal/benign disease, three of nine grade I tumours, four of 12 grade II tumours, but 11 of 11 grade III tumours. Mean levels were higher in grade III tumours ($P < 0.0001$). When the levels of 72 kDa collagenase and its active 62 kDa form were considered together, there was no difference between the benign and malignant cases ($P = 0.55$), but the amount of active enzyme, considered as a proportion of the 62 + 72 kDa forms, was significantly higher in malignant disease ($P = 0.003$). There was also a trend towards a higher proportion of active enzyme with increasing tumour grade ($P < 0.0001$). *In situ* hybridisation and immunohistochemistry studies showed that that mRNA and protein for the 92 kDa enzyme was primarily found in the tumour stroma. mRNA for the 72 kDa enzyme was also found in stromal areas. This study demonstrates a clear relationship between production of Type IV collagenases and malignant breast disease. Inhibitors of these enzymes may be of value in preventing metastatic disease.

In normal glandular tissue a specialised form of extracellular matrix, the basement membrane, separates epithelial cells from the underyling stroma. In benign breast disease and carcinoma *in situ*, basement membrane is preserved, but is partially or completely lost in invasive carcinoma of the breast (Siegel *et al.*, 1981). Basement membrane contains laminin, heparan sulphate proteoglycan and type IV collagen and other extracellular matrix components. The degradation of type IV collagen may be a prerequisite for metastasis formation.

Matrix metalloproteases are a family of highly homologous proteolytic enzymes involved degradation of basement membrane. Each member has a different substrate specificity. Matrix metalloprotease-1 (MMP-1) degrades interstitial collagen, stromelysin (MMP-3) degrades proteoglycans, whilst the two type IV collagenases of molecular weights 72 kDa (MMP-2) and 92 kDa (MMP-9) are capable of degrading type IV collagen, gelatin and fibronectin (Liotta & Stetler-Stevenson, 1991). Type IV collagenases are secreted in an inactive proenzyme form and activation results from the removal of an 80 amino acid sequence from the N-terminus (Stetler-Stevenson *et al.*, 1989). This can be achieved *in vitro* by treatment with trypsin or organomercurials but the precise sequence of events required for activation *in vivo* is unknown. Matrix metalloproteinases and other proteolytic enzymes such as cathepsins and plasminogen activating factors may be involved (Yagel *et al.*, 1989; Goldberg *et al.*, 1990; Ward *et al.*, 1991).

There is much experimental evidence linking type IV collagenase expression with the metastatic phenotype. Increasing tumour cell secretion of type IV collagenase by transfecting with oncogenes, or by clonal selection, enhances their metastic behaviour in experimental animals and their ability to invade a reconstituted basement membrane *in vitro* (Garbisa *et al.*, 1987, 1988). Similarly, decreasing type IV collagenase activity by tissue inhibitor of metalloprotease, TIMP, reduces the metastatic capacity of tumour cells in animal models (Alvarez *et al.*, 1990; DeClerk *et al.*, 1992).

Several recent studies have reported type IV collagenases in human tumour biopsies. The 72 kDa enzyme was found in neoplastic, but not normal, colonic epithelia by immunohistochemical methods (Levy *et al.*, 1991). Pyke *et al.* (1992), demonstrated expression of mRNA for the 72 kDa enzyme by fibroblasts adjacent to tumour cell islands in basal and squamous cell carincoma, but found no expression in the tumour cells. This same study demonstrated mRNA for the 92 kDa enzyme by macrophages and some tumour cells in skin tumours, but not in normal skin. Previous studies of type IV collagenase expression in human breast neoplasms have been restricted to immunohistochemical studies using polyclonal (Daidone *et al.*, 1991; Monteagudo *et al.*, 1990) antisera to the 72 kDa type IV collagenase. These studies demonstrated that 72 kDa type IV collagenase was expressed in both normal and malignant breast biopsies, chiefly by the myoepithelial cells (Daidone *et al.*, 1991; Monteagudo *et al.*, 1990). The disadvantage of immunohistochemistry is that while it gives useful information concerning the localisation of enzymes, levels cannot be accurately quantified. It has not been possible to distinguish between the inactive pro-enzyme and the activated type IV collagenase. Collagenolytic assays are insensitive and require large amounts of tissue. Additionally they cannot detect inactive proforms of metalloproteinases. The presence of tissue inhibitors of matrix metaloproteinases (TIMPS) further complicate interpretation of results.

In this study, we have used zymography (gel substrate analysis) to investigate the levels of type IV collagenases in breast tumours. In this technique, small fragments of homogenised tissue are loaded onto polyacrylamide gels impregnated with gelatin. Collagenase present in the tissue digests the gelatin, leaving a clear band after the gel is stained for protein. Zymography can distinguish between the 92 and 72 kDa enzymes and can also distinguish between inactive and active forms of the 72 kDa enzyme, because SDS causes enzyme activation (Birkedal-Hansen & Taylor, 1982). The 72 kDa enzyme appears on the zymogram as a band of apparent molecular weight 72 kDa (inactive precursor) with a doublet beneath this of molecular weight 59/62 kDa (activated enzyme with inhibitory N-terminal sequence removed). The active 81 kDa form of the 92 kDa enzyme is not resolved on the gels and the enzyme appears as a single band. With computer assisted image analysis, zymography can be used as a quantitative analytical method.

Combined with quantitative analysis of active and inactive type IV collagenases in benign and malignant breast disease, we have localised the RNA for the 92 and 72 kDa collagenases using *in situ* hybridisation. We have also localised the 92 kDa protein using immunohistochemistry.

Correspondence: B. Davies.
Received 13 October 1992; and in revised form 21 December 1992.

### Methods

*Tissue samples*

Breast tissue removed at excision biopsy or mastectomy for breast disease was cryopreserved in liquid nitrogen. Three cases of normal breast tissue, eight of benign breast disease, and 32 of invasive carcinoma of varying histological grade were examined. Tumour grading was by the modified Bloom-Richardson classification (Elston, 1984). A 5 µm thick section was cut from a face area of each tumour. Samples were homogenised in 50 µl of SDS-PAGE sample buffer containing glycerol (10% v/v) SDS (1% w/v) and bromophenol blue using treff microhomogenisers (Scott Lab). Adjacent sections were cut and used for protein estimation, immunohistochemistry and *in situ* hybridisation. Sections were cut at varying depths in the block to assess reproducibility of zymography.

*Gelatin zymography*

Gelatinolytic zymography was performed as described by Brown *et al.*, 1990. This technique can distinguish between the 72 and 92 kDa type IV collagenases. Additionally, the method can detect the inactive proforms of collagenases because SDS causes activation of the enzymes without proteolytic cleavage of the inhibitory N-terminal sequence (Birkedal-Hansen & Taylor, 1982). Homogenised samples (50 µl) were applied directly without heating or reduction to a 5% w/v stacking polyacrylamide gel laid over an 11% (w/v) polyacrylamide gel containing $1\ mg\ ml^{-1}$ gelatin and 0.1% (w/v) SDS. Gels were run at room temp at 180 V for 3.5 h. After incubation of gels in 2.5% Triton X-100 for 30 min to remove SDS, the gels were incubated for 16 h at 37°C in 50 mM Tris-HCl, pH 7.6 containing 0.2 M NaCl, 5 mM CaC12 and 0.02 w/v Brij-35. Gels were stained for 3 h in 30% methanol/10% glacial acetic acid containing 0.5% (w/v) Coomassie Brilliant Blue G 250 and destained in the same solution in the absence of dye.

The amount of collagenase present affects not only the intensity of the band produced but also the size of the band. Conventional linear densitometric analysis is therefore inadequate for assessment of collagenolytic activity. We have used computer assisted image analysis to overcome this problem. Images of wet gels were captured using a Sony DXC-151P video camera connected to capture hardware/software (Screen Machine/SM-camera). NIH Image 1.43 software equipped with gel plotting macros was used to measure the integrated density of each band. Conditioned media from human melanoma RPMI 7951 cells and from TPA stimulated HT1080 fibrosarcoma cells were used as type IV collagenase standards (Brown *et al.*, 1990; Weinberg *et al.*, 1990). RPMI 7951 constituitively secretes 72 kDa type IV collagenase and the activity (Lane 1, Figure 1) present in 20 µl of conditioned media (as detected by zymography) was defined as 100 arbitrary units of type IV procollagenase. Conditioned media from TPA stimulated HT 1080 fibrosarcoma cells also contained 72 kDa procollagenase (Lane 2, Figure 1). In addition to the activated forms of this enzyme (59 kDa/62 kDa doublet). HT-1080 cells also expressed the 92 kDa type IV collagenase (Lane 1, Figure 1) and the activity contained in 20 µl of conditioned media was defined as 100 arbitrary units of this enzyme. The resolution of 11% acrylamide gels is insufficient to distinguish between the pro-form of 92 kDa type IV collagenase and its 81 kDa activated form.

*Protein estimation*

Single 5 µm cryostat sections from each tumour were homogenised in 1% (w/v) SDS and diluted 10-fold in water before measuring protein content against bovine serum albumin using a Bio-Rad protein assay reagent (Bradford, 1976).

In situ *hybridisation*

Antisense 72 kDa and 92 kDa was generated from the pGEM3-72K and pGEM3-92K (kindly provided by British Biotechnology, Oxford, UK) using the relevant RNA polymerase (Promega Biotech, Madison, USA). The negative control was sense RNA generated from the same vector linearised in the opposite direction. *In vitro* transcriptions were performed using Promega Biotech transcription kits to incorporate $^{35}$S-UTP (Amersham International SJ 1303). Restriction enzymes were all obtained from Pharmacia.

*In situ* hybridisation was carried out on cryostat sections as in Naylor *et al.*, 1990.

*Immunohistochemistry*

A sheep polyclonal antibody to Mr 92 kDa collagenase was a generous gift of Dr Gillian Murphy, Strangeways Laboratory, Cambridge. This antibody has been extensively characterised (Murphy *et al.*, 1989). Following blocking in normal rabbit serum, the primary antibody was applied in a 1/200 dilution. The second layer was peroxidase conjugated rabbit anti-goat (Dakopatts) which had been pre-absorbed in normal human serum. Sections were developed using 0.01% $H_2O_2$/Diaminobenzidine-tetrachloride and counterstained with Mayer's haemotoxylin. Parallel runs omitting the primary antibody were included as a negative control in all cases.

**Figure 1** Zymography of breast cancer tissue. Lane 1 = 12 µl RPMI 7951 supernatant showing 72 kDa type IV collagenase. Lane 2 = 12 µl TPA stimulated HT-1080 supernatant showing 92 kDa type IV collagenase, 72 kDa type IV collagenase and its activated forms (59/62 kDa doublet). Lanes 3–12 = breast cancer samples A–J.

### Results

### *Zymography*

Type IV collagenase activity was assessed in 11 cases of normal/benign breast disease and 32 cases of invasive ductal carcinoma of varying grade, by measuring their gelatinolytic activity in gel substrate analysis. For each tumour sample the activities of 92 kDa and 72 kDa type IV collagenases were calculated in arbitrary units per 10 µg of protein. The activity of the 72 kDa type was resolved into its inactive proform (72 kDa) and its active form (62 kDa). Conditioned media from human tumour cell lines, which are known to contain type IV collagenases, were used as standards as described in the Methods.

Tables I and II show the levels of different species of type IV collagenases in benign and malignant breast tissue of varying tumour grade. Computer assisted image analysis was used to quantify the results. To assess the reproducibility of the quantification technique, sections were cut from different portions from the same tumour and collagenase activity assessed. Variation in collagenase activity between sections of the same tumour was a mean of 11.3% in 18 determinations.

The 92 kDa form of type IV collagenase and its activation products were present in three of 11 benign cases, three of nine grade I tumours, four of 12 grade II tumours but in 11 of 11 grade III tumours (Table I). Mean levels of this form were significantly higher in grade III tumours when compared with the other cases (32.25 U ± 10.67 *vs* 5.19 U ± 1.98, $P < 0.0001$). Using this percentage of acrylamide gel we were unable to accurately distinguish between 92 kDa procollagenase and its activation product of Mr 81 kDa (Brown *et al.*, 1990).

Table II shows the levels of 72 kDa proform and the activated 62 kDa collagenase in the breast biopsies. The 72 kDa enzyme was found in all benign and malignant tissues examined. The 62 kDa active form was found in 10/11 normal/benign, 7/9 grade I, 12/12 grade II, and 11/11 grade III tumours. When the levels of 72 kDa proenzyme and its active 62 kDa form were considered together there was no difference between the benign and malignant cases (30.05 U ± 4.84 *vs* 27.64 U ± 3.48, $p = 0.55$). When the inactive (72 kDa) form was considered alone, there were significantly higher levels in benign compared with malignant disease (22.88 U ± 3.71 *vs* 14.04 U ± 1.89, $P = 0.03$). The mean level of the active (62 kDa) form was higher in malignant compared with benign samples (13.57 U ± 1.94 *vs* 7.18 U ± 2.17), though this failed to reach standard levels of significance ($P = 0.06$). When the amount of the active (62 kDa) enzyme was considered as a proportion of the total amount of the 72 + 62 kDa species present, the proportion in malignant disease was significantly higher than that found in benign/normal breast tissue (0.45 *vs* 0.20, $P = 0.003$). Although the total amount of 72 + 62 kDa activity did not differ significantly between tumours of different histological grades, the proportion of the active form of the enzyme correlated with tumour grade, test for trend (rank correlation), $P < 0.0001$ (Table II).

Figure 1 shows an example of zymograms from ten cases of primary breast cancer (lanes A–J). All of these tumours express the Mr 72 kDa type IV procollagenase. These activities ranged from 4.2 U (sample F) to 37.9 U (sample A). Levels of the Mr 62 kDa enzyme varied between 0U (sample F) to 26.2 U (sample A). Considerable variation was observed in the levels of the Mr 92/81 kDa enzyme (185.4 U sample C to 0U samples, D,E,F,G).

**Table I** Levels of 92 kDa collagenase in breast biopsies

| *Pathology* | *Number of positive cases/total* | *Mean 92 + 81 kDa activity (U/10 µg protein ± S.E.)* |
|---|---|---|
| Benign/ normal | 3/11 | 6.58 ± 3.4 |
| Malignant | | |
| grade I | 3/9 | 1.34 ± 0.8 |
| grade II | 4/12 | 6.80 ± 4.27 |
| grade III | 11/11 | 32.29 ± 10.67 |

Homogenates of tissue were applied to 11% acrylamide gels as described in the Methods.
Levels of 92 kDa collagenase in grade III tumours *vs* levels in all other tumour/benign specimens $P < 0.0001$.

### *Localisation of type IV collagenase expression*

Further experiments were carried out to localise these enzymes in the tumour tissue. Figure 2a shows the location of the 92 kDa collagenase mRNA in a grade III ductal carcinoma. mRNA expression was seen in collections of elongated spindle shaped cells lying within tumour stroma. These cells were of fibroblast or macrophage morphology. The signals were particularly strong in stroma adjacent to foci of ductal carcinoma *in situ*. Protein expression was also confirmed in adjacent sections using a polyclonal antibody to the 92 kDa form of the enzyme. Distribution of protein was shown to be similar to that of the mRNA (Figure 2b). As well as stromal distribution within spindle shaped cells, 92 kDa mRNA and protein was also identified in putative myoepithelial cells in some areas of ductal carcinoma *in situ* adjacent to the invasive carcinoma (Figure 2c and d). Consistent results were obtained in ten samples of malignant breast disease.

Detection of mRNA for the 72 kDa enzyme by *in situ* hybridisation was also carried out in seven cases. This enzyme showed a similar pattern of expression to the 92 kDa enzyme, being found predominantly in spindle shaped cells in the stroma.

**Table II** Levels of 72 kDa collagenase and its 62 kDa form in breast biopsies

| *Pathology* | *No:* | *Mean collagenase activity (U/10 µg protein)* 62 kDa | 72 kDa | 62 + 72 | *Ratio* 62/62 + 72 | |
|---|---|---|---|---|---|---|
| Benign/ Normal | 11 | 7.18 | 22.88 | 30.05 | 0.20 | |
| Malignant | | | | | | |
| grade I | 9 | 14.69 | 17.58 | 32.27 | 0.33 | test for |
| grade II | 12 | 11.48 | 13.84 | 25.4 | 0.45 | trend |
| grade III | 11 | 14.93 | 11.36 | 26.29 | 0.55 | $P < 0.0001$ |

Homogenates of tissue were applied to 11% acrylamide gels as described in the Methods. The 72 kDa proform was found in all tissues. The 62 kDa active form was found in 10/11 normal/benign, 7/9 grade I, 12/12 grade II, and 11/11 grade III samples.
Levels of 62 kDa benign *vs* malignant $P = 0.06$.
Levels of 72 kDa benign *vs* malignant $P = 0.03$.
Ratio of 62 to 62 + 72 kDa benign *vs* malignant $P = 0.003$.

**Figure 2** Localisation of 92 kDa collagenases in malignant breast tissue. Adjacent sections showing *in situ* hybridisation to mRNA **a** and protein **b** for the 92 kDa collagenase in stomal cells of a Grade 3 ductal carcinoma. Adjacent sections showing *in situ* hybridisation to mRNA **c** and protein **d** for the 92 kDa collagenase in myoepithelial cells surrounding a grade 1 ductal carcinoma *in situ*. T-tumour cells. Tcis-areas of carcinoma *in situ*. MY-myoepithelial cell. MA-cell of macrophage-like morphology.

### Discussion

In this study increased production of 92 kDa type IV collagenase was associated with increasing severity of grade of human breast carcinomas. *In situ* hybridisation and immunostaining showed that mRNA and protein for this enzyme were expressed not by the tumour cells themselves but by cells in the surrounding stroma. Some of the cells expressing the 92 kDa enzyme were myoepithelial cells, other cells present in the stroma also expressed the enzyme and were probably macrophages or fibroblasts. Cells of the mononuclear phagocyte lineage have been widely reported to secrete 92 kDa type IV collagenase (Hibbs *et al.*, 1987; Welgus *et al.*, 1990) and this activity is affected by, for example, treatment with LPS or concanavalin A. The tissue location of macrophages also affects the extent to which they express this enzyme, but the precise control mechanisms and mediators operating *in vivo* have not yet been elucidated. Expression of the 72 kDa type IV collagenase by fibroblasts *in vitro* and *in vivo* is well documented (Seltzer *et al.*, 1981; Goldberg *et al.*, 1986; Ballin *et al.*, 1988) but they do not secrete the 92 kDa enzyme under normal conditions. However SV40 transformed fibroblasts do secrete the 92 kDa enzyme (Wilhelm *et al.*, 1989) and we speculate that fibroblasts may be induced to express this enzyme by tumour derived factors.

The control of type IV collagenase secretion by cytokines and growth factors is poorly defined. TGF-$\beta$1 increases secretion of both the 72 kDa and 92 kDa enzymes in tumour cells and fibroblasts (Overall *et al.*, 1989; Weinberg *et al.*, 1990; Welch *et al.*, 1990) and TNF-$\alpha$ has been reported to increase the secretion of the 92 kDa enzyme, but not the 72 kDa type, by tumour cells (Brenner *et al.*, 1989; Okada *et al.*, 1990). Interestingly, we have found that a minority ($<0.1\%$) of predominantly stromal cells in a series of 80 breast cancer biopsies express mRNA for TNF and produce immunoreactive TNF protein. Phenotyping of cells in sequential sections suggests that CD68 positive activated macrophages produce this cytokine. The level of TNF expression increased with severity of histological grade (Miles *et al.*, manuscript in preparation).

Seventy-two kDa type IV collagenase has previously been reported to be localised in tumour epithelial cells and in myoepithelial cells in normal breast and tumour (Monteagudo *et al.*, 1990). We found no correlation between disease severity and levels of expression of 72 kDa type IV collagenase. However when the proportion of activated to total 72 kDa type IV collagenase was compared to tumour grade, there was a clear correlation with ratio increasing in proportion to grade. Immunohistochemical methods would not have been able to detect this relationship. Seventy-two kDa type IV collagenase, in common with other matrix metalloproteinases, is secreted in inactive pro-form and activation occurs by proteolytic removal of an N-terminal sequence (Stetler-Stevenson *et al.*, 1989). This can be achieved *in vitro* by treatment with trypsin or by autoproteolysis following treatment with organomercurial compounds. How this proteolytic activation occurs *in vivo* and how it is controlled is unclear. However, exogenously added 72 kDa enzyme is activated by concanavalin A treated fibroblasts and this activation is blocked by inhibitors of protein synthesis. Activation is also blocked by inhibitors of matrix metalloproteinases (Ward et al., 1991). We need to understand further the activation process of the 72 kDa enzyme in order to explain our finding that this occurs more readily in tumours of higher histological grade. Investigations of the microenvironment of breast tumours, particularly in terms of endogenous cytokine/growth factor production, are also required to understand control of type IV collagenase expression.

As the collagenases localise to the stroma, changes in levels with tumour grade may be related to differences in stromal development. This explanation is, however, unlikely for two reasons. First, we find not only a change in level of 92 kDa collagenase, but also a higher proportion of grade III tumours expressing the enzyme; and second, we find a significant increase in the ratio of active to total 72 kDa enzyme with increasing tumour grade although the total amount of enzyme is not increased.

The relationship between type IV collagenase expression and tumour grade may partly explain the relationship between tumour grade and behaviour since basement membrane degradation is a prerequisite for invasion and metastasis. Inhibitors of Type IV collagenase activity could, therefore, find a role in the management of breast cancer and clinical studies are indicated.

We wish to thank Dr Peter Brown and Dr Alan Galloway from British Biotechnology Ltd for providing the cDNA probes for collagenases, and for useful discussion. Dr Gillian Murphy, Strangeways Laboratory, Cambridge, kindly provided the antibody to the 92 kDa enzyme. We are also indebted to Mr George Holt of the Photographic Department, Imperial Cancer Research Fund, for help with image analysis, Walter Gregory, Clinical Oncology Unit, Guys Hospital, for help with statistical analysis.

### References

ALVAREZ, O.A., CARMICHAEL, D.F. & DE CLERCK, Y.A. (1990). Inhibition of collagenolytic activity and metastasis of tumour cells by a recombinant human tissue inhibitor of metalloproteinases. *J. Natl Cancer Inst.*, **82**, 589–595.

BALLIN, M., GOMEZ, D.E., SINHA, C.C. & THORGEIRSSON, U.P. (1988). Ras oncogene mediated induction of a 92 kDa metalloproteinase; strong correlation with the malignant phenotype. *Biochem. Biophys. Res. Commun.*, **154**, 832–838.

BIRKEDAL-HANSEN, H. & TAYLOR, R.E. (1982). Detergent-activation of latent collagenase and resolution of its component molecules. *Biochem. Biophys. Res. Commun.*, **107**, 1173–1178.

BRADFORD, M.M. (1976). A rapid and sensitive method for the quantitation of microgram quantities of protein utilizing the principle of protein-dye binding. *Anal. Biochem.*, **72**, 248–254.

BRENNER, D.A., O'HARA, M., ANGEL, P., CHOJKIER, M. & KARIN, M. (1989). Prolonged activation of jun and collagenase genes by tumour necrosis factor-a. *Nature*, **337**, 661–662.

BROWN, P.D., LEVY, A.T., MARGULIES, I.M.K., LIOTTA, L.A. & STETLER-STEVENSON, W.G. (1990). Independent expression and cellular processing of Mr 72,000 type IV collagenase and interstitial collagenase in human tumourigenic cell lines. *Cancer Res.*, **50**, 6184–6191.

DAIDONE, M.G., SILVESTRINI, R., D'ERRICO, A., DI FRONZO, G., BENINI, E., MANCINI, A.M., GARBISA, S., LIOTTA, L.A. & GRIGIONI, W.F. (1991). Laminin receptors, collagenase IV and prognosis in node-negative breast cancers. *Int. J. Cancer*, **48**, 529–532.

DECLERCK, Y.A., PEREZ, N., SHIMADA, H., BOONE, T.C., LANGLEY, K.E. & TAYLOR, S.M. (1992). Inhibition of invasion and metastasis in cells transfected with an inhibitor of metalloproteinases. *Cancer Res.*, **52**, 701–707.

ELSTON, C.W. (1984). The assessment of histological differentiation in breast cancer. *Aust. N.Z. J. Surg.*, **54**, 11–15.

GARBISA, S., POZZATTI, R., MUSCHEL, R.J., SAFFIOTTI, U., BALLIN, M., GOLDFARB, R.H., KHOURY, G. & LIOTTA, L.A. (1987). Secretion of type IV collagenolytic protease and metastatic phenotype: induction by transfection with c-Has-ras but not c-Ha-ras plus Ad2-Ela. *Cancer Res.*, **47**, 1523–1528.

GARBISA, S., NEGRO, A., KALBIC, T., POZZATTI, R., MUSCHEL, R., SAFFIOTTI, U. & LIOTTA, L.A. (1988). Type IV collagenolytic activity linkge with the metastatic phenotype induced by ras transfection. *Adv. Exp. Med. Biol.*, **233**, 179–186.

GOLDBERG, G.I., WILHELM, S.M., KRONBERGER, A., BAUER, E.A., GRANT, G.A. & EISEN, A.Z. (1986). Human fibroblast collagenase. Complete structure and homology to an oncogene transformation-induced rat protein. *J. Biol. Chem.*, **261**, 6600–6605.

GOLDBERG, G.I., FRISCH, S.M., HE, C., WILHELM, S.M., REICH, R. & COLLIER, I.E. (1990). Secreted proteases. Regulation of their activity and their possible role in metastasis. *Ann. N. Y. Acad. Sci.*, **580**, 375–384.

HIBBS, M.S., HOIDAL, J.R. & KANG, A.H. (1987). Expression of a metalloproteinase that degrades native type V collagen and denatured collagens by cultured human alveolar macrophages. *J. Clin Invest.*, **80**, 1644–1650.

LIOTTA, L.A. & STETLER-STEVENSON, W.G. (1991). Tumour invasion and metastasis: an imbalance of positive and negative regulation. *Cancer Res.*, **51**, 5054s–5059s.

LEVY, A.T., CIOCE, V., SOBEL, M.E., GARBISA, S., GRIGIONI, W.F., LIOTTA, L.A. & STETLER-STEVENSON, W.G. (1991). Increased expression of the Mr 72,000 type IV collagenase in human colonic adenocarcinoma. *Cancer Res.*, **51**, 439–444.

MONTEAGUDO, C., MERINO, M.J., SAN-JUAN, J., LIOTTA, L.A. & STETLER-STEVENSON, W.G. (1990). Immunohistochemical distribution and type IV collagenase in normal, benign and malignant breast tissue. *Am. J. Pathol.*, **136**, 585–592.

MURPHY, G., WARD, R., HEMBRY, R.M., REYNOLDS, J.J., KUHN, K. & TRYGGVASON, K. (1989). Characterisation of gelatinase from pig polymorphonuclear leucocytes. A metalloproteinase resembling type IV collagenase. *Biochem. J.*, **258**, 463–472.

NAYLOR, M.S., STAMP, G.W.H. & BALKWILL, F.R. (1990). Investigation of cytokine gene expression in human colorectal cancer. *Cancer Res.*, **50**, 4436–4440.

OKADA, Y., TSUCHIYA, H., SHIMIZU, H., TOMITA, K., NAKANISHI, I., SATO, H., SEIKI, M., YAMASHITA, K. & HAYAKAWA, T. (1990). Induction and stimulation of a 92 kDa gelatinase/type IV collagenase production in osteosarcoma and fibrosarcoma cell lines by tumour necrosis factor-a. *Biochem. Biophys. Res. Commun.*, **171**, 610–617.

OVERALL, C.M., WRANA, J.L. & SODEK, J. (1989). Independent regulation of collagenase, 72 kDa progelatinase and metalloproteinase inhibitor expression in human fibroblasts by transforming growth factor-b. *J. Biol. Chem.*, **264**, 1860–1869.

PYKE, C., RALKIAER, E., HUHTALA, P., HURSKAINEN, T., DANO, K. & TRYGGVASON, K. (1992). Localization of messenger RNA for Mr 72,000 and 92,000 type IV collagenases in human skin cancers by *in situ* hybridization. *Cancer Res.*, **52**, 1336–1341.

SELTZER, J.L., ADAMS, S.A., GRANT, G.A. & EISEN, Z.A. (1981). Purification and properties of a gelatin-specific neutral protease from human skin. *J. Biol. Chem.*, **256**, 4662–4668.

SIEGAL, G.P., BARSKY, S.H., TERRANOVA, V.P. & LIOTTA, L.A. (1981). Stages of neoplastic transformation of human breast tissue as monitored by dissolution of the basement membrane components. *Invasion Metastasis*, **1**, 54–65.

STETLER-STEVENSON, W.G., KRUTZSCH, H.C., WACHNER, M.P., MARGUILES, I.M.K. & LIOTTA, L.A. (1989). The activation of human type IV collagenase proenzyme. Sequence identification of the major conversion product following organomercurial activation. *J. Biol. Chem.*, **264**, 1353–1356.

WARD, R.V., ATKINSON, S.J., SLOCOMBE, P.M., DOCHERTY, A.J.P., REYNOLDS, J.J. & MURPHY, G. (1991). Tissue inhibitor of metalloproteinases-2 inhibits the activation of 72 kDa progelatinase by fibroblast membranes. *Biochim. Biophys. Acta*, **1079**, 242–246.

WEINBERG, W.C., BROWN, P.D., STETLER-STEVENSON, W.G. & YUPSA, S.H. (1990). Growth factors specifically alter hair follicle cell proliferation and collagenolytic activity alone or in combination. *Differentiation*, **45**, 168–178.

WELCH, D.R., FABRA, A. & NAKAJIMA, M. (1990). Transforming growth factor-b stimulates mammary adenocarcinoma cell invasion and metastatic potential. *Proc. Natl Acad. Sci.*, **87**, 7678-7682.

WELGUS, H.G., CAMPBELL, E.J., CURY, J.D., EISEN, A.Z., SENOIR, R.M., WILHE;M, S.M. & GOLDBERG, G.I. (1990). Neutral metalloproteinases produced by human mononuclear phagocytes. *J. Clin. Invest.*, **86**, 1496-1502.

WILHELM, S.M., COLLIER, I.E., MARMER, B.L., EISEN, Z.A., GRANT, G.A. & GOLDBERG, G.I. (1989). SV40-transformed human lung fibroblasts secrete a 92 kDa type IV collagenase which is identical to that secreted by normal human macrophages. *J. Biol. Chem.*, **264**, 17213-17221.

YAGEL, S., WARNER, A.H., NELLANS, H.N., LALA, P.K., WAGHORNE, C. & DENHARDT, D.T. (1989). Suppression by cathepsin L inhibitors of the invasion of amnion membranes by murine cancer cells. *Cancer Res.*, **49**, 3553-3557.